# The influence of soil dry-out on the record-breaking hot 2013/2014 summer in Southeast Brazil

**DOI:** 10.1038/s41598-022-09515-z

**Published:** 2022-04-07

**Authors:** J. L. Geirinhas, A. C. Russo, R. Libonati, D. G. Miralles, P. M. Sousa, H. Wouters, R. M. Trigo

**Affiliations:** 1 Instituto Dom Luiz (IDL), Faculdade de Ciências, Universidade de Lisboa, 1749-016 Lisbon, Portugal; 2 Departamento de Meteorologia, Universidade Federal do Rio de Janeiro, Rio de Janeiro, 21941-919 Brazil; 3 Hydro-Climate Extremes Lab (H-CEL), Ghent University, Ghent, Belgium; 4 Instituto Português do Mar e da Atmosfera (IPMA), 1749-077 Lisbon, Portugal; 5 Environmental Modelling Unit, Flemish Institute for Technological Research, Mol, Belgium

**Keywords:** Atmospheric dynamics, Climate change, Hydrology, Natural hazards

## Abstract

The 2013/2014 summer in Southeast Brazil was marked by historical unprecedented compound dry and hot (CDH) conditions with profound socio-economic impacts. The synoptic drivers for this event have already been analyzed, and its occurrence within the context of the increasing trend of CDH conditions in the area evaluated. However, so far, the causes for these record temperatures remain poorly understood. Here, a detailed characterization of the 2013/2014 austral summer season over Southeast Brazil is proposed, emphasizing the role played by land–atmosphere interactions in temperature escalation. We demonstrate that a strong soil moisture–temperature coupling regime promoted record-breaking temperatures levels exceeding almost 5 °C over the previous highest record, and played a key role in triggering an outstanding ‘mega-heatwave’ that lasted for a period of around 20 days. This pronounced soil desiccation occurred within a current climate change trend defined by drier and hotter conditions in the region. The soil dry-out, coupled with strong radiative processes and low entrainment of cooler air masses through mesoscale sea-breeze circulation processes, led to a water-limited regime and to an enhancement of sensible heat fluxes that, ultimately, resulted in a sharp increase of surface temperatures.

## Introduction

The global warming trend has led to the recent occurrence of historically unprecedented heatwaves and record-breaking temperatures^1,2^. These mega-heatwave episodes have been responsible for a massive number of heat-related deaths^3^, high levels of air pollution from severe wildfires^4^, peaks in energy consumption^5^, exacerbation of drought events^6^, and reduced crop yields^7^. Several studies have shown that the escalation of temperatures during recent episodes in Europe could not be explained by atmospheric circulation anomalies alone, and that the combined effect of local soil dryness and high heat advection is a necessary ingredient^6,8–10^. Soil desiccation leads to a reduction in the evaporative cooling and an increase in the sensible heat flux between surface and atmosphere^11^. A more complex effect of soil moisture on temperature was identified by Ref.^6^ for the 2010 Russian mega-heatwave, when the observed temperature anomalies were triggered by horizontal heat advection and warming from soil dry-out conditions, combined with a progressive entrainment of warm and dry air from higher levels of the atmosphere into to the atmospheric boundary layer, also driven by drying soils. This effect is not just local, as heatwaves can also propagate through horizontal heat advection, fueled by upwind soil drought^12^. Such compound dry and hot (CDH) conditions were also recently observed in North America^13^, Asia^14,15^ and Australia^16^. Future climate projections suggest that events with a magnitude similar to the recent mega-heatwaves will become the norm by the end of the century^2,17,18^. This is in part due to critical changes in precipitation and evaporation leading to transitions from energy-limited to water-limited regimes, increasing the likelihood of CDH events^19,20^.

Despite recent efforts to understand the occurrence of CDH extremes, particularly on the mid-latitude regions of the Northern Hemisphere^12,21–23^, the Southern Hemisphere region still lacks a similar detailed analysis of these compound conditions. To the best of our knowledge, in what concerns South America, and specifically Brazil, only recently a few preliminary assessments started to be undertaken^24–27^. Moreover, certain regions of Southeast Brazil (SEB) have been experiencing a clear increase in the number and severity of heatwaves and droughts over the last decades^28–30^, and positive trends in the number of CDH events^25^. The historically unprecedented drought conditions recorded during the austral summer seasons (December to February) of 2013/2014 and 2014/2015 have contributed substantially to the drying trend recently observed in SEB^25,31,32^. The inhabitants of the metropolitan region of São Paulo, the fourth most populated megacity in the world^33^, faced a dramatic water supply crisis as a result of this severe drought during the 2013/2014 summer^34^. The water scarcity led to serious shortages in agricultural irrigation and in energy production from hydropower plants.

Several studies have looked into this extreme summer to identify the large-scale and synoptic conditions leading to the occurrence of the drought and hot event^31,35–37^; others have investigated how this season fits into an increasing trend of CDH conditions in the area^25^. However, so far, none of these assessments has explored and quantified the long-term record nature of the observed hot conditions and their true spatial extent. The critical role played by land–atmosphere interactions and by strong soil moisture–atmosphere coupling conditions in temperature escalation remains poorly described. In addition, little attention has been given to assessing the mesoscale atmospheric processes that triggered the warm conditions observed in some of the urban areas of SEB, such as the urban areas of São Paulo (UASP) and Curitiba (UACT), where heat-stress levels are known to cause critical impacts in public health^33,38^.

In light of climate projections, which point to a warmer and drier future in the region^39–41^, it is therefore crucial to enhance the knowledge about the key processes and feedbacks associated with such extreme compound events. Here, we aim to quantify in detail the exceptionality of the warm temperature levels experienced during the 2013/2014 austral summer season over SEB, and analyze the role played by land–atmosphere interactions in temperature escalation. We also pretend to assess, in a high spatial resolution, the mesoscale atmospheric mechanisms that triggered the outstanding near-surface temperature anomalies over SEB.

## Results

### The historically unprecedented hot and dry 2013/2014 summer season

**Figure 1:**
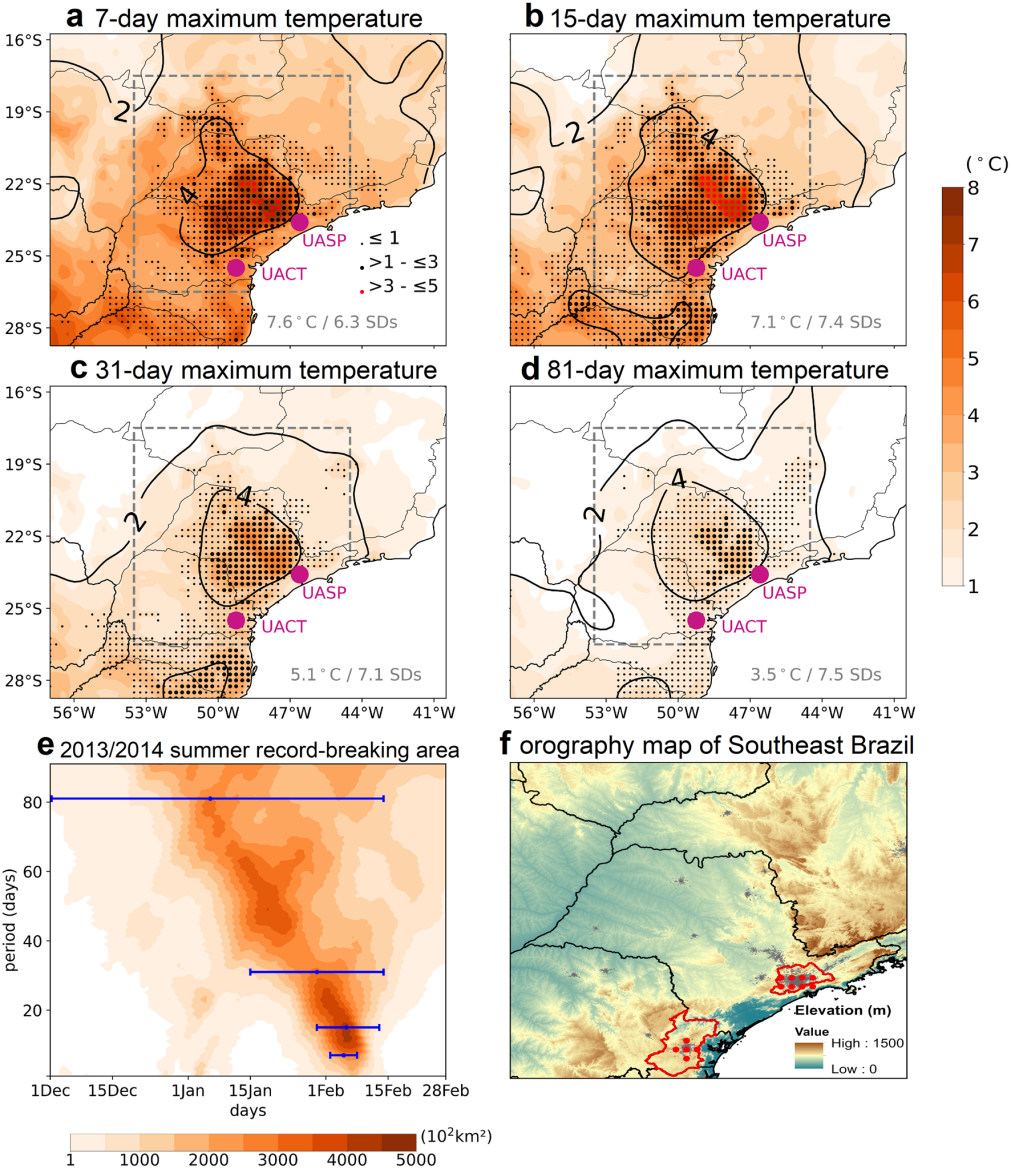
Spatio-temporal characterization of the record-breaking 2013/2014 summer. Maximum surface temperature anomalies (°C, relative to 1981–2010) during the 2013/2014 summer for 7-day (**a**), 15-day (**b**), 31-day (**c**) and 81-day average periods (**d**). Contour lines depict the anomaly divided by the corresponding standard deviation of all summer days of the reference period. The dots highlight record-breaking temperature anomalies with the size and the color being proportional to the exceedance over the previous period. The magenta dots indicate the location of the UASP and UACT. The maximum temperature anomaly is shown in the bottom right corner. Temporal evolution of the spatial extent (in 10^2^ km^2^) of areas experiencing record-breaking temperatures at different time scales during the 2013/2014 summer (**e**). Only the grid points within the grey box shown in the previous panels are considered. Blue bars indicate the period of maximum extension for the time scales represented in the previous panels. Orography map of the region within the grey box (**f**). The limits of the UASP and the UACT are shown by the red polygons. The grey shade highlights the urbanized areas and the red dots indicate the ERA5 and GLEAM grid-points considered to compute area averages for these particular urban areas.

During the 2013/2014 summer season, SEB witnessed exceptional surface warm conditions at different temporal scales, ranging from weekly to seasonal (Fig. 1a–d). The state of São Paulo was the center of the highest maximum of temperature anomalies for all temporal scales. The anomalies for this particular region exceeded the mean by 4 standard deviations for all the temporal scales, underlining the massive amplitude and persistence of the induced temperature extremes. The strongest anomalies were observed for the 15-day average periods (up to 8 °C in some areas), with most of the state of São Paulo witnessing historically unprecedented hot temperatures, sometimes representing an exceedance of almost 5 °C over the previous highest record. The area covered by record-breaking temperatures for all temporal scales (Fig. 1) extended from the state of São Paulo towards more southern regions. Figure 1e displays the temporal evolution during the 2013/2014 summer of the spatial extent of areas, within the grey box depicted in Fig. 1a-d, experiencing record-breaking temperatures. For shorter time scales, temperature records were established during two distinct periods of the summer season: the first, less intense, took place from the end of December until the first half of January; the second, much stronger, developed from the end of January until the first half of February. For the 15-day period time scale, February 5th was the day witnessing the highest area with record-breaking temperatures, with around 450,000 km^2^. It is important to stress that this value underestimates the real spatial extent of the warm conditions since it was restricted to the area within the grey box. Finally, the record-breaking pattern was not symmetric in time, indicating that the warm conditions that started in mid-January ceased abruptly after mid-February (Fig. 1e).

It is important to acknowledge that the data from the ERA5 reanalysis datasets used here, only goes back to 1980 (see “Data and methods” section), which undermines the statistical significance of these record-breaking temperature conditions. Thus, we also used a long-term daily maximum temperature record since 1933, from the University of São Paulo meteorological station, located within the city of São Paulo (see “Data and methods” section). This much longer time series allowed us to observe that, in fact, the 2013/2014 summer witnessed the highest temperature since 1933 for different temporal scales (Supplementary Fig. S1). Although this result was computed for a single point over SEB, it confirms the analysis obtained using the reanalysis datasets for the region and provides additional reliable information on the true temporal extent of these outstanding warm conditions.

**Figure 2:**
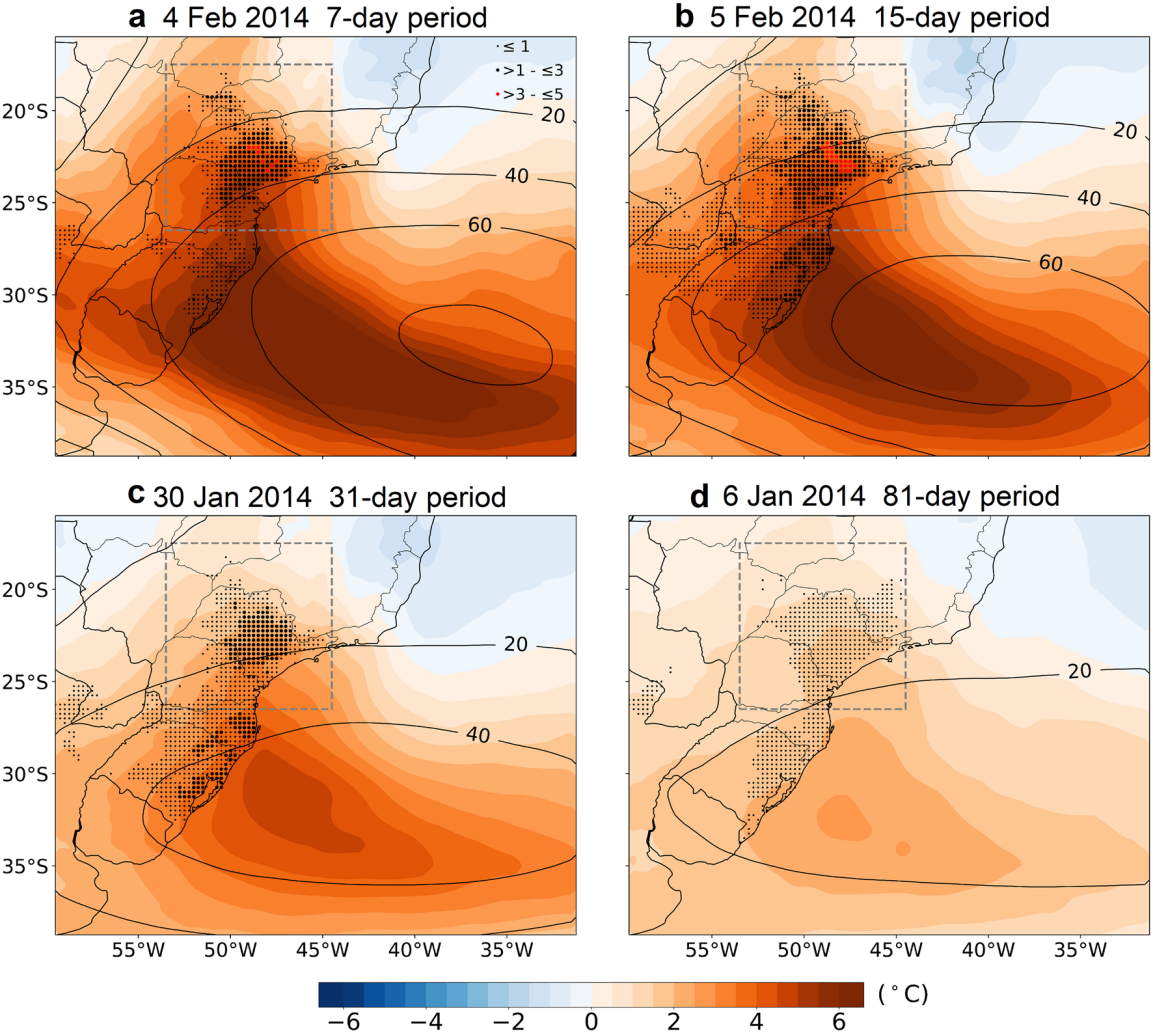
Characterization of the synoptic conditions for the 2013/2014 summer during the days having a maximum area covered by record-breaking temperatures at different temporal scales. Shading shows the spatial distribution of the 850-hPa temperature (°C) anomalies and contours the spatial distribution of the 500-hpa geopotential height (gpm) anomalies (relative to the 1981–2010 period) for 7-day (**a**), 15-day (**b**), 31-day (**c**) and 81-day average periods (**d**) centered on the day indicate at the top of each panel. The dots highlight the grid-points with record-breaking land surface temperature anomalies during the respective average periods. The size and the color are proportional to the exceedance over the previous period.

The synoptic analysis conducted for this summer shows that such record-breaking temperatures were triggered by quasi-stationary anticyclonic circulation anomalies over the eastern branch of south Atlantic Ocean, near the southeast coast of Brazil (Fig. 2). This high-pressure configuration favors the escalation of temperatures in the region due to a combination of mechanisms, including diabatic heating, strong subsidence and warm air advection^10,42,43^. Figure 2 shows the anomalous atmospheric pattern observed during the days with the largest spatial extension affected by record-breaking temperatures for the four temporal scales considered in Fig. 1e (see blue lines). In particular, time scales compatible with synoptic disturbances (7 and 15 day) clearly show that a high-pressure system was established in the region. Such quasi-stationary circulation anomaly led to a strong adiabatic heating mechanism that was particularly intense over the coastal land section of SEB and over the state of São Paulo, where most of the record-breaking temperature values were observed. This finds support in the spatial signature of the 850-hPa temperature anomalies that shows a slight westward shift regarding the pressure anomaly center and a pronounced continental penetration towards these land areas.

**Figure 3:**
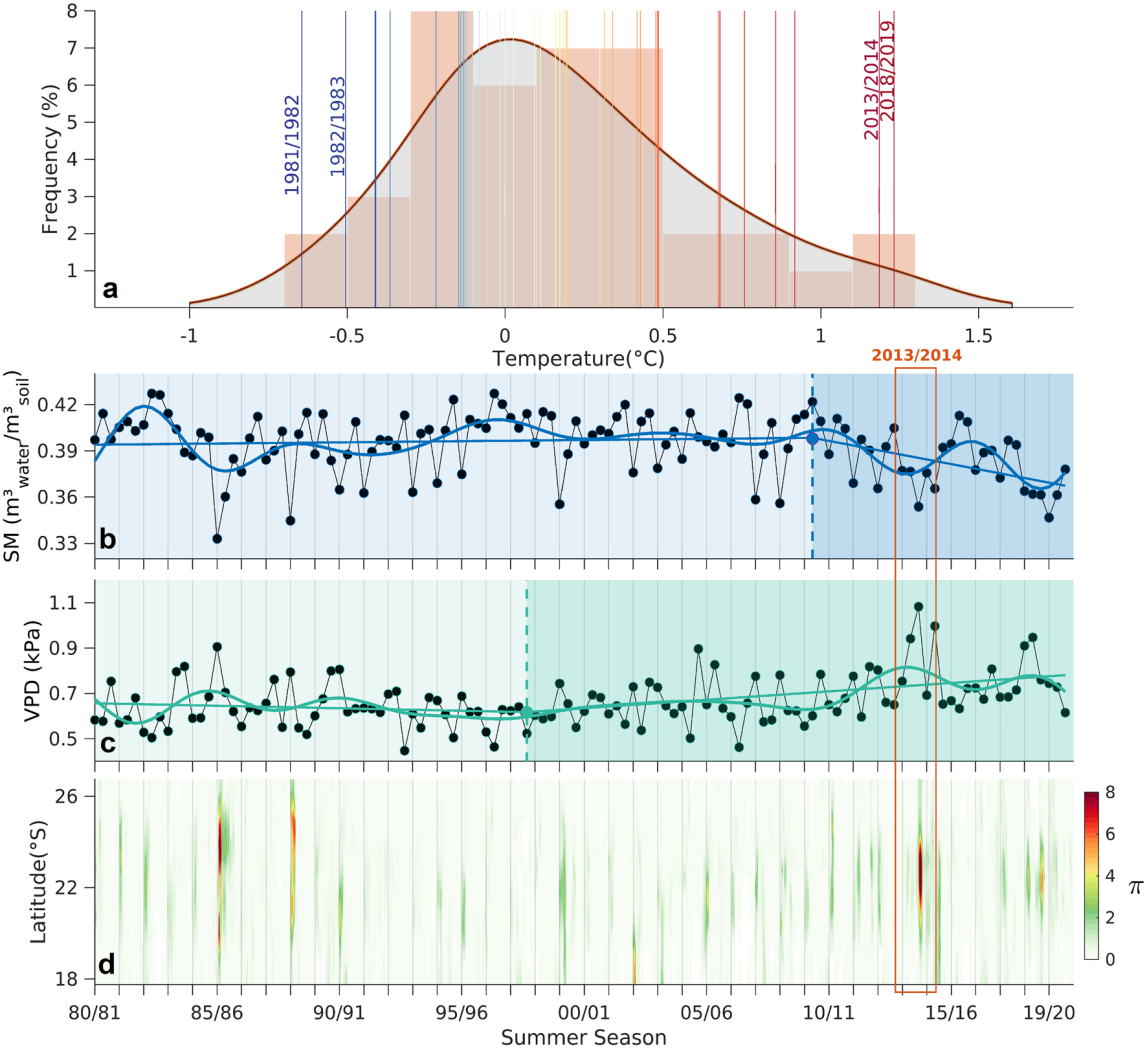
Analysis of the extreme summer hot and dry conditions during the 2013/2014 summer in a historical evolution perspective. Kernel distribution function for the average summer temperature anomalies (°C) from 1980 to 2020 observed for the region within the grey box shown in Figs. 1 and 2 (**a**)*.* The vertical colored lines indicate the mean surface temperature anomaly values for each summer season. Historical evolution from 1980 to 2020 of monthly mean summer soil moisture (${m}_{water}^{3}/{m}_{soil}^{3}$) and vapor pressure deficit (kPa) values (**b, c**, respectively). The bold lines result from the application of a 10-year low-pass Lanczos filter and a linear regression model with two segmented (i.e., piece-wise) linear relationships separated by a break point (obtained from an iterative process described in Supplementary Material) highlighted by the filled colored dot. The monthly mean values result from area averages applied for the region within the grey box in Figs. 1 and 2. Mean daily π coupling metric per each 0.25° latitude within the grey box shown in Figs. 1 and 2, for the summer seasons from 1980 to 2020 (**d**).

The 2013/2014 season was, at the time, the hottest ever recorded summer for the region bounded by the grey box in Figs. 1 and 2 (Fig. 3a), only recently surpassed by the 2018/2019 summer. In fact, the four hottest summers were recorded during the short 8-year period from 2013 to 2020, reflecting a pronounced warming trend in the last decade of the analysis period. Regarding soil moisture, one can observe that the values recorded, although not being record-breaking, were extremely low during the 2013/2014 summer (Fig. 3b). Such dry conditions occurred within a pronounced decreasing trend of the mean monthly summer soil moisture levels (− 0.032 ${m}_{water}^{3}/{m}_{soil}^{3}$ per decade, statistically significant at a 5% level) that started during the 2009/2010 summer (breakpoint obtained from an iterative process described in Supplementary Material) and has contributed, since then, to a total estimated mean monthly decrease of 0.036 ${m}_{water}^{3}/{m}_{soil}^{3}$. This can be explained, in part, by the occurrence of higher evaporation rates supported by the recent summer warming trend (Fig. 3a). On the other hand, the extreme hot conditions experienced during the 2013/2014 summer triggered record-breaking vapor pressure deficit (VPD) values, indicating the high evaporative demand observed during this period and how the low soil moisture was partially due to large evaporative rates (Fig. 3c). Similarly to soil moisture, the outstanding VPD observed during this summer season occurred within an increasing trend of the mean monthly summer VPD for the region. Such trend started in 1997/1998 summer (0.096 kPa per decade, statistically significant at a 5% level) and, since then, has contributed to a total estimated mean monthly increase of 0.2208 kPa. As a result of such concurring soil desiccation, enhanced evaporative demand and severe warm conditions, a strong soil moisture–temperature coupling was observed in the region during this summer (Fig. 3d), indicating that when the hot temperature anomalies occurred a strong soil moisture deficit was present, leading to a large flux of sensible heat from surface to the atmosphere. In fact, such soil moisture–temperature coupling levels only find parallel in the values recorded during the summer of 1985/1986.

This reinforces the interest of analyzing in detail the exceptional concurring conditions of extreme heat, soil desiccation and strong soil moisture–atmosphere coupling witnessed during the 2013/2014 summer.

### The outstanding 2013/2014 summer in the UASP and UACT in a historical context

**Figure 4:**
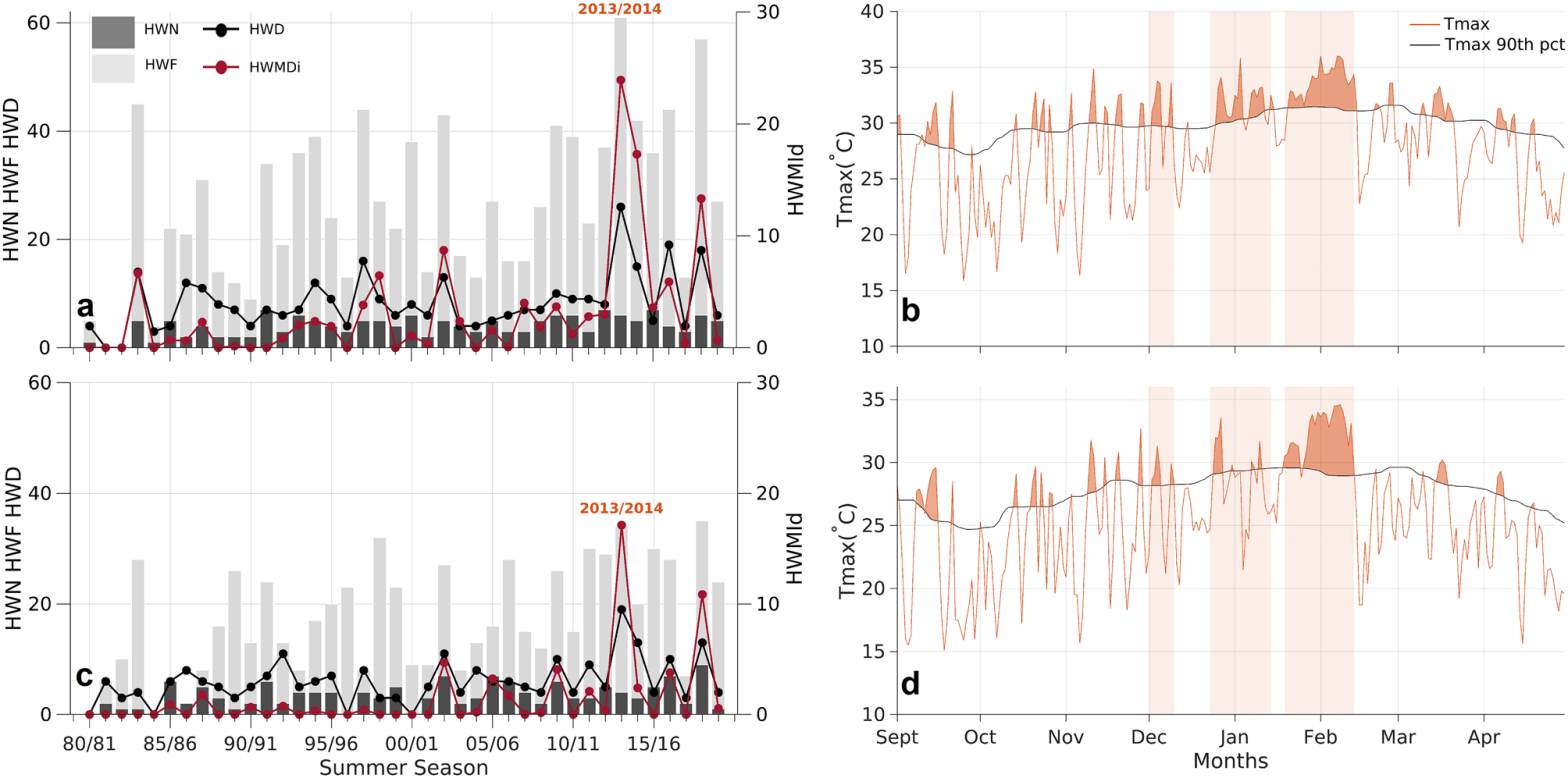
Analysis of the heatwave conditions over the UASP and UACT. Temporal evolution for the summers from 1980 to 2020 of the heatwave parameters: HWN, HWF, HWD and HWMId (see “Data and methods” section) for the UASP (**a**) and UACT (**c**). Daily maximum temperature values (°C, orange line) and the respective 90th calendar day climatological (1981–2010 reference period) percentile (black line) from September 2013 to April 2014 over the UASP (**b**) and UACT (**d**). The results for the UASP were obtained using the averages between the daily maximum temperature values observed at two meteorological stations located within the UASP (see “Data and methods” section). The results for the UACT were also obtained using a maximum temperature record from a single meteorological station located within the UACT (see Data and Methods).

The historical evolution of several summer heatwave parameters from 1980 to 2020 reveals that extreme temperatures were particularly experienced over the UASP and UACT during the 2013/2014 summer season (Fig. 4). For the UASP, the maximum HWF (number of summer days under a heatwave regime) was observed during this period, reaching 61 days. Regarding the UACT, this summer witnessed the second highest HWF with 35 days, a value that was only shortly exceeded by the one recorded during the 2018/2019 summer (36 days). The longest ever recorded heatwave (HWD) was also observed for both urban areas during the 2013/2014 summer season, when the UASP (UACT) was affected by an outstanding episode that lasted for 26 (19) consecutive days. Similarly, the highest value of the heatwave magnitude index daily (HWMId) was observed during this period, revealing the unprecedented magnitude of this heatwave. Moreover, the 2013/2014 summer was also subject to the occurrence of several hot spells with an almost perfect temporal match between cities, despite them being more than 300 km apart from each other (Fig. 1f). Three hot periods (grey shaded areas in Fig. 4b,d) were defined by grouping several heatwaves separated by short periods of mild temperatures. Accordingly, the first hot period occurred during the first days of December; the second from the end of December to mid-January; and the third corresponds to a mega-heatwave episode, from mid-January to mid-February. These two last hot periods identified for both urban areas correspond to those previously identified in Fig. 1e when analyzing the areas covered by record-breaking temperatures. In fact, the days having the highest land extension covered by record-breaking temperatures when considering 7-day (February 4th), 15-day (February 5th) and 31-day (January 31st) average periods are all included in this massive mega-heatwave identified for both urban areas. It is important to note that these results, obtained from in situ meteorological data are in agreement with the previous analysis obtained using the ERA5 datasets. This indicates once again that the data from the reanalysis model is reliable to reconstruct the warm conditions of this summer, particularly for the urban areas considered here.

### Soil moisture–temperature coupling during the 2013/2014 summer

**Figure 5:**
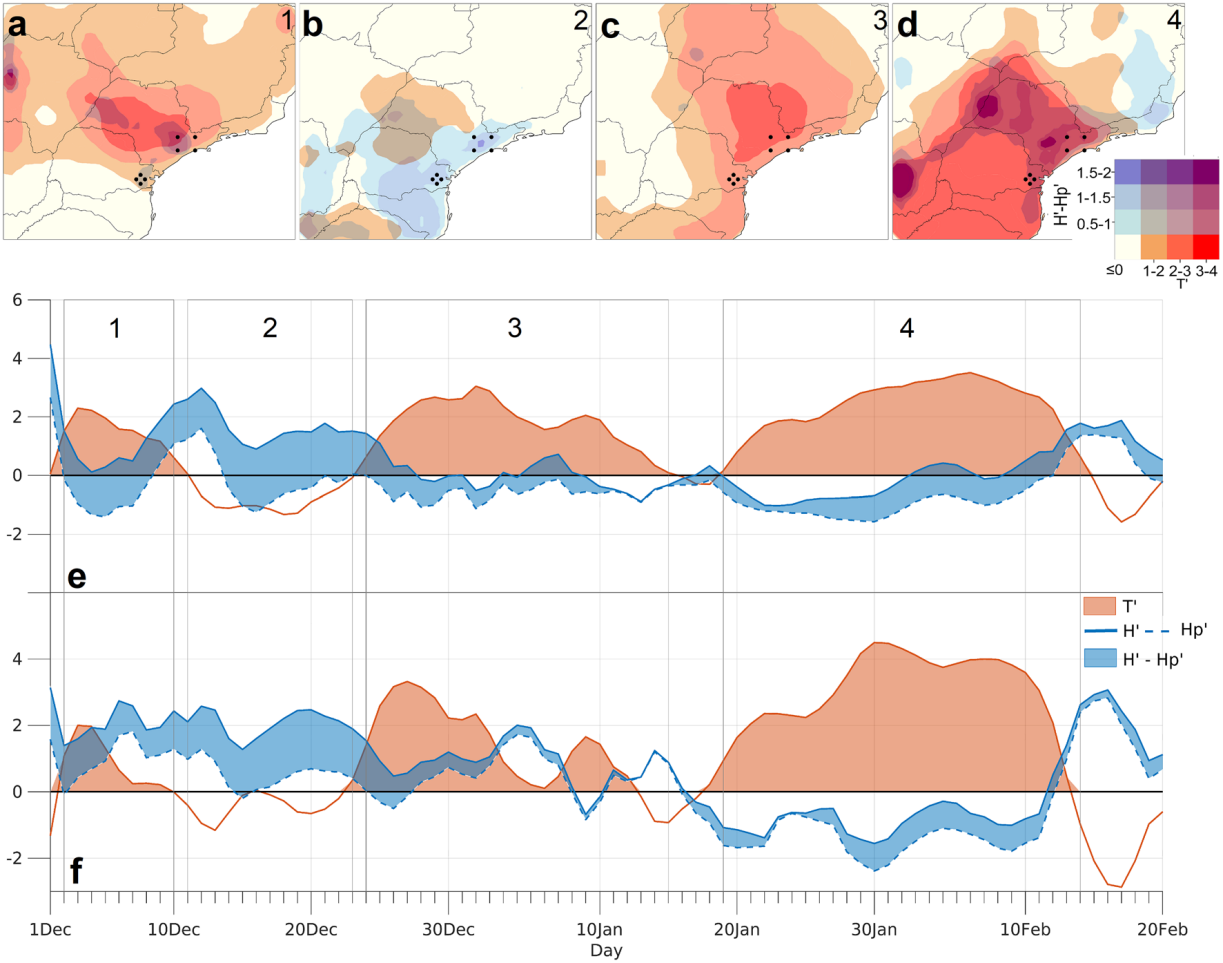
Soil moisture–temperature coupling during the 2013/2014 summer. Spatial distribution over SEB of the temperature and energy coupling anomalies throughout the four previously defined periods within the 2013/2014 summer season (**a–d**) and chronologically defined in **e, f** by grey boxes. Time series for the 2013/2014 summer season of spatial average values of the temperature and energy coupling terms over the UASP (**e**) and UACT (**f**). Black dots on the top panels mark the geographical limits of these two urban areas. The location of the model grid-points considered for the computation of these area averaged time series for the two urban areas is shown in Fig. 1f.

The spatial signature over SEB and the temporal evolution for the UASP and UACT of the two soil moisture–temperature coupling terms analyzed here (Tʹ: temperature term; ${H}^{{\prime}}-{H}_{p}^{{\prime}}$: energy term) reveals that several independent periods were defined by distinct anomalies in both terms (Fig. 5). In fact, the occurrence of these anomalies matches the hot periods identified and discussed previously for these urban areas in Fig. 4, which suggests an influence of land–atmosphere feedbacks on temperature anomalies. Accordingly, four periods were defined: (i) from December 1st to 9th (corresponding to the first hot period defined during the analysis of Fig. 4), (ii) from December 11th to 22nd, (iii) from December 24th to January 14th (which corresponds to the second hot period) and (iv) from January 19th to February 13th, corresponding to the mega-heatwave episode. During the first period, some SEB regions, and particularly the UASP and UACT, were marked by positive values of both terms (Fig. 5a,e,f). This is indicative of a high coupling regime (i.e. high π; see Supplementary Fig. S2), in which the temperature anomalies are influenced by a pronounced evaporative stress linked to a strong soil desiccation and large amounts of shortwave radiative energy available at surface (i.e., large values of ${H}^{{\prime}}-{H}_{p}^{{\prime}}$). During the second period (Fig. 5b,e,f), large values of the energy term concurred with negative temperature anomalies, indicating that although dry conditions and large amounts of incoming radiative energy at surface concurred, air temperature (likely driven by advection of cooler air masses) was not anomalously positive, pointing to a low coupling regime (i.e. low π; see Supplementary Fig. S2).

During the third period (Fig. 5c,e,f), relatively low soil moisture–temperature coupling conditions were maintained (Supplementary Fig. S2), although they were explained by an opposite behavior in what concerns the contributions of the temperature and energy terms. By contrast to the preceding days, positive temperature anomalies concurred with a relatively low energy term, indicating that although the atmosphere warmed, the soil moisture restriction relaxed. This was evident for the UASP and UACT, and resulted from the occurrence of brief precipitation events (Supplementary Fig. S3). Finally, the fourth period, corresponding to the mega-heatwave event, was marked by a positive contribution from the energy and temperature terms throughout most of SEB and particularly over its central region, where the warmest conditions were recorded (Figs. 1, 3d). This triggered a strong coupling regime over the UASP, UACT and the north and northwestern surrounding regions (Fig. 5d–f, Supplementary Fig. S2).

Therefore, the observed record-breaking temperature anomalies and the outstanding magnitude of this mega-heatwave were somewhat driven by a pronounced soil moisture imbalance that forced the surface to start delivering part of the available radiative energy back to the atmosphere through sensible heat flux. This suggests that dry conditions were likely the extra ingredient that defined this episode as a historically unprecedent mega-heatwave rather than a regular heatwave with shorter duration and less intense temperature anomalies.

### Mesoscale meteorological drives of heatwave conditions over the UASP and UACT

**Figure 6:**
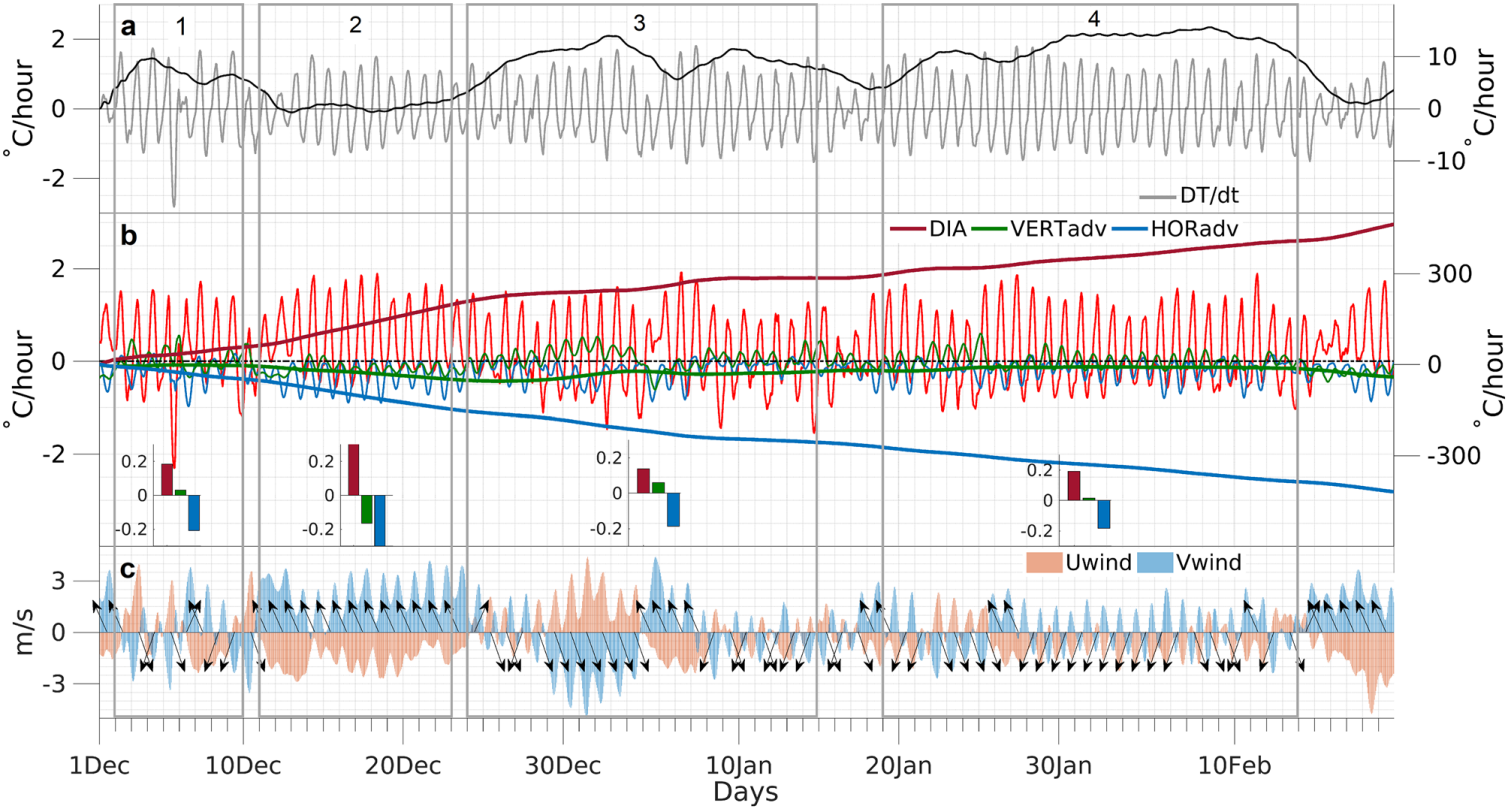
Atmospheric mesoscale characterization of the 2013/2014 summer over the UASP. Time series of area average values computed (at an hourly scale) for the UASP and throughout the 2013/2014 summer season of several regional high resolution meteorological parameters. 925-hPa (local near-surface) temperature variation rate (grey line) and respective cumulative values (accumulated over time, black line) (**a**). Contribution of the diabatic term (red), of vertical (green) and horizontal (blue) temperature advection terms for the observed 925-hpa temperature variation rate (see “Data and methods” section) (**b**). Ticker lines indicate the cumulative values. The inset bar plots show the mean hourly contribution of each mechanism during each one of the four previously defined periods within the summer season. Time series of zonal (Uwind) and meridional (Vwind) wind components (colors) (**c**). Arrows indicate the daily predominant wind direction. The location of the model grid-points considered for the computation of these area averaged time series for the UASP is shown in Fig. 1f.

In order to disentangle the mesoscale atmospheric mechanisms that triggered such anomalies in temperature and land–atmosphere coupling over the UASP and UACT, we zoom in the heatwave event at hourly time scales. The hourly evolution of area-averaged values in near-surface temperature variation, the contribution of the diabatic processes, and horizontal and vertical temperature advection to this temperature variation was assessed for the UASP (Fig. 6) and UACT (Supplementary Fig. S4). These area-averaged values were computed considering the ERA5 model grid points located within both urban areas (highlighted by the red dots in Fig. 1f). The heat-stress conditions observed during the first hot period over the UASP, were triggered by a pronounced atmospheric heating rate during the first days of December 2013 (Fig. 6a). This was the outcome of a positive balance between the contribution of the diabatic term (positive) and the horizontal temperature advection term (negative), explaining the positive energy coupling term depicted in Fig. 5, which highly depends on the available shortwave radiative energy at the surface. During the second period (encompassing the days defined by a temperature cooling between the first and second hot periods—see Figs. 4a, 5e), a pronounced atmospheric cooling (see black line in Fig. 6a) was observed. This resulted from a strong negative contribution of both horizontal and vertical temperature advection terms, combined with a positive contribution of local radiative processes (Fig. 6b). This can be observed by analyzing the consistent decreasing trend in the cumulative values of vertical and horizontal temperature advection (green and blue bold lines in Fig. 6b) and the mean hourly negative contribution of these two processes to the temperature variation (bar plots in Fig. 6b). Thus, although clear sky conditions were maintained, the lower troposphere suffered an intense cooling due to a continental penetration of oceanic air masses. The influence throughout the UASP of this cooler air coming from the South Atlantic is evidenced by the observed values of the zonal and meridional wind components (Fig. 6c), that indicate the dominant presence of southeasterly winds (see black arrow in Fig. 6c). Therefore, although diabatic heating remained, explaining the positive energy coupling term (Fig. 5b,e), the coupling temperature term was negative, resulting in a week soil moisture–atmosphere coupling over the UASP. The third period was marked again by a pronounced atmospheric heating that occurred mostly between the last days of December and the first days of January (Fig. 6a), which was supported by a dominant positive contribution of diabatic processes and of vertical temperature advection mechanisms from December 28th to January 4th (see tick and bold green lines in Fig. 6b). Such intense contribution from the vertical temperature advection term is associated with the predominance of north and northwesterly winds (Fig. 6c). Analyzing the orography of the UASP and of the surrounding regions (Fig. 1f), it is possible to observe that these north and northwesterly offshore winds brought air masses from more elevated regions towards the UASP, generating an adiabatic air compression mechanism responsible for a near-surface heating. These conditions concurred with relatively lower negative contribution of horizontal temperature advection (blue line in Fig. 6b) and relatively lower diabatic contribution when compared to other periods. Thus, although clear-sky conditions were present, part of the atmospheric heating process resulted from katabatic winds explaining the previously identified relatively low (high) energy (temperature) coupling term for the region during this period (Fig. 5c,e).

Finally, during the fourth period, strong positive coupling conditions were observed for the UASP (Fig. 5). The mega-heatwave event recorded during this period was generated by a pronounced atmospheric heating from January 19th to 22nd and later from January 27th to 31st (Fig. 6a). These periods of temperature escalation were strongly promoted by local diabatic processes and by residual contributions from the vertical and horizontal advection mechanisms. Thus, clear-sky conditions coupled with a relatively low entrainment of cooler oceanic air masses towards the UASP triggered the observed temperature anomalies. In fact, Supplementary Fig. S5 shows that the region of interest within SEB (highlighted by the grey rectangle) as well as both urban areas, was not affected by the advection of warmer air masses from remote regions. When analyzing the anomaly wind pattern, one may conclude that the observed wind configuration during both the whole summer season and during the mega-heatwave episode was anomalously eastward (Supplementary Fig. S5c,f), promoting the advection towards the UASP, UACT and the surrounding regions of slightly colder air masses (Supplementary Fig. S5a,b,d,e). On the other hand, Supplementary Fig. S6 shows that the anomalies of the surface net solar radiation over this area were remarkably positive during both periods, indicating that the diabatic contribution was always the dominant mesoscale mechanism fueling the temperature variation. Such strong contribution of radiative processes for the temperature escalation explains the high energy coupling term as well as the high temperature coupling term identified for the region (Fig. 5d,e), with the last one receiving an extra boost due to a breaking of the sea-land breeze mesoscale regime.

The corresponding analysis for the UACT (Supplementary Fig. S4) is very similar. Due to the smoothed orography of the UACT and of the surrounding regions (Fig. 1f), the vertical temperature advection mechanisms were always residual, and so, the near-surface temperature variation was mainly controlled by the balance resulting from the diabatic and the horizontal temperature advection processes.

## Discussion and conclusions

During the outstanding 2013/2014 summer season, SEB experienced a historical unprecedented heatwave. It was the longest and most severe summer heatwave episode ever recorded over both the UASP and UACT for the past four decades, and was responsible for an increase in the numbers of heat-related mortality^38,44^. The obtained high HWMId values for this season revealed the exceptional magnitude of this particular event that occurred during a record-breaking summer season, which finds parallel in its magnitude and extent with the remarkable 2003 European and 2010 Russian summers^2,45,46^.

The hot periods recorded over SEB concurred with pronounced drought conditions which were already described in recent studies^25,31,34,35,37^. Here we show that the occurrence of dry surface conditions, triggered by higher VPD and lower precipitation levels^25^ has been increasing for the region during the last decade. A permanent soil moisture decreasing trend in the near-future could lead soil moisture to reach values lower than the so-called critical level^11^. Accordingly, this would enhance the role played by the surface in constraining evaporation and in influencing the land energy and water balances. In this context, there is an increased likelihood for the occurrence of strong soil moisture–temperature coupling conditions such as the one described during this 2013–2014 event. Supplementary Figure S7 shows the difference of the correlation values obtained between soil moisture and the evaporative fraction for two sub-periods encompassing, respectively, the summers for the 1981–2000 and 2001–2020 periods. One can observe that in some regions of SEB (namely the center of São Paulo state), the correlation coefficient values have increased significantly between the early period (1981–2000) and the latter period (2001–2020). This highlights that soil moisture has been gaining more influence in the portitioning of incoming energy to latent and sensible heat fluxes. However, we acknowledge that some caution must be taken due to the short period considered, and therefore this particular topic deserves further analysis. Nevertheless, this process is something already expected to occur in some regions of the globe under several climate change scenarios^19,20^.

Previous studies have shown that the observed long-term precipitation deficit and the severe high temperatures observed in SEB were induced due to a suppression of the South Atlantic Convergence Zone^31^, which agrees with the quasi-stationary anticyclonic pattern identified over SEB and highlighted here in Fig. 2. These synoptic conditions were triggered by anomalous convective activity in the equatorial sections of both the Indian and Pacific oceans near Australia^31,35,36^, that imposed a large perturbation in the tropical zonal Walker cell and in the extratropical meridional Hadley cell, establishing a stationary Rossby wave spanning from west Pacific to South Atlantic Ocean. The eastern signature of this large wave pattern was the occurrence of the quasi-stationary anticyclone structure identified in the present work (Fig. 2).

The analysis of the soil moisture–temperature coupling terms and of the mesoscale atmospheric mechanisms that led to the near-surface temperature increase over the UASP and the UACT, revealed that the relationship between the soil dryness conditions and heatwaves was marked by distinct phases. Although dry conditions were present during almost the entire summer season, a positive soil moisture–temperature coupling, leveraged by enhanced diabatic heating processes and a suppression of the normal atmospheric cooling by sea breezes, was only observed during two distinct periods, being one the discussed mega-heatwave. Thus, the observed concurring drought conditions were important for the amplification and maintenance of this mega-heatwave through the establishment of a water-limited regime and an increase in the sensible heat flux between surface and atmosphere. The high values obtained for the soil moisture–temperature coupling over SEB were similar to the ones obtained for the 2003 European and 2010 Russian mega-heatwaves^6,9^ which indicates the historical relevance of this episode.

Although several previous studies have already characterized the main synoptic drives for this summer season^31,35–37^, to the best of our knowledge, none had explored and quantified in detail the exceptionality of the induced warm conditions in such a high temporal and spatial scale. Therefore, our results and conclusions highlighted a chapter about this historical summer season that remained so far unexplored, by showing that the observed record-breaking warm conditions weren’t explained by synoptic circulation anomalies alone and that land–atmosphere feedbacks and their inter-links with mesoscale processes played a crucial role.

Useful metrics to perform a thorough characterization and quantification of the magnitude of CDH events could also be drawn using the results presented here. This would allow for a more robust comparison between these compound episodes throughout periods defined by a climate change context. Moreover, they would represent a guideline for predicting future episodes of this kind and mitigate the associated natural, socio-economic and public health impacts for Brazil. The increased heat-related impact observed during the 2013/2014 summer^38,44^ should encourage political, health and civil protection authorities to seek tools and mitigation measures to improve the control of illnesses related to hot periods, particularly in megacities in developing countries like Brazil^33,47^. Finally, robust projections indicate a future climate scenario controlled by hotter and drier conditions in South America and specifically in Brazil^39–41^ and consequently, by an increasing frequency of more intense and longer lasting CDH episodes.

## Data and methods

### Data

Daily maximum temperature (hereafter, Tmax) and precipitation data from two meteorological stations located within the UASP were used. The first station (23.50° S, 46.63° W) belongs to the Brazilian National Institute of Meteorology (INMET) and was used to assess the daily precipitation record for the UASP from 1980 to 2020. The second station (23.65° S, 46.62° W) belongs to the University of São Paulo and provides a long-term Tmax record from 1933 to 2020. Accordingly, the Tmax analyzed for the UASP and for the period from 1980 to 2020, resulted from daily averages between the Tmax values observed in these two meteorological stations. On the other hand, the daily precipitation record analyzed for the UASP belongs to the INMET station only. Regarding the UACT, precipitation and Tmax data from an INMET station (23.50° S, 46.63° W) were used for the period from 1980 to 2020. Other daily meteorological data, including surface net radiation, geopotential height, near-surface temperature, as well as temperature and zonal and meridional wind at several pressure levels, were extracted from the European Centre of Medium-range Weather Forecast (ECMWF) ERA5 reanalysis (Copernicus Climate Change Service, C3S)^48^. Daily surface variables including soil moisture and evaporation were extracted from the Global Land Evaporation Amsterdam Model (GLEAM v3.3a)^49,50^. Both ERA5 and GLEAM data share a 0.25° × 0.25° horizontal resolution. Anomalies were computed with respect to the climatological seasonal cycle (1981–2010).

### Record-breaking temperature definition

To identify record-breaking temperature anomalies in a particular summer, we define the respective historical period which contains all the summer days (December to January) from 1980 up to the year of the given event. In order to identify record-breaking temperature values we adopted, similarly to Ref.^45^, the following rationale: (1) the running means of daily anomalies for different time scales centered on each summer day are calculated, allowing superposition (e.g., for a 5-day time scale and for February 6th the period to be considered for the running mean goes from February 4th to February 8th); (2) for each grid-point and time-scale, the maximum value of the $n\times 90$ sample is retained as historical maximum, with *n* being the number of years from 1980 to the year before the summer season in consideration and 90 corresponding to the total number of days within the summer season; (3) a record-breaking temperature is identified if the maximum anomaly for a given summer period surpasses the historical maximum for the corresponding temporal scale. For instance, for a 5-day time scale and for a specific grid-point, if the highest temperature anomaly recorded for the summer season of 2013/2014 is higher than the maximum anomaly ever recorded for the historical period (containing the $n\times 90$ summer days from 1980 up to the 2013/2014 summer), than this particular grid-point experienced during the 2013/2014 summer a record-breaking temperature anomaly. The data used for these calculations corresponded to the daily mean near-surface temperature from ERA5 reanalysis.

### Heatwave definition

Heatwaves were defined as periods of three or more consecutive days with daily Tmax values above the climatological (1981–2010) 90th percentile, calculated based on a 15-day moving window centered in the specific calendar day^25,51^. Hot periods were defined by grouping several heatwaves separated by short periods of heat-stress relief. Based on this criterion, the heatwave incidence per summer season (December–February, 1980–2020) was explored by assessing the values of several heatwave parameters: the number of heatwave episodes (HWN), the sum of participating heatwave days (HWF), and the length (in days) of the longest heatwave event (HWD). To account for both heatwave duration and intensity, values of the heatwave magnitude index daily (HWMId)^2^ were also computed for each summer season (more information regarding the HWMId metric can be found in the Supplementary Material). The data used for these calculations correspond to in situ daily Tmax records from two meteorological stations located within the UASP and UACT.

### Soil moisture–temperature coupling

The π diagnostic proposed by Ref.^9^, was used to assess and quantify the magnitude of soil moisture–temperature coupling. This metric estimate two terms based on near surface air temperature (*T*), evaporation (*E*), potential evaporation (${E}_{p}$) and surface net radiation (${R}_{n}$). π is defined as the product of a temperature term (${T}^{\prime}$) and an energy term (${H}^{{\prime}}-{H}_{p}^{{\prime}}$):

1 $$\pi =\left({H}^{{\prime}}-{H}_{p}^{{\prime}}\right) {T}^{{\prime}},$$

where *H* quantifies the actual sensible heat resulting from the estimate evaporation and surface net radiation levels, and ${H}_{p}$ quantifies the sensible heat that would occur assuming potential evaporation:

2 $$\left({H}^{{\prime}}-{H}_{p}^{{\prime}}\right)={\left({R}_{n}-\lambda E\right)}^{{\prime}}-{\left({R}_{n}-\lambda {E}_{p}\right)}^{{\prime}}.$$

${T}^{{\prime}}$, ${H}^{{\prime}}$ and ${H}_{p}^{{\prime}}$ indicates, respectively, the daily anomalies of *T*, *H* and ${H}_{p}$ expressed in the number of standard deviations relative to their expectation, and *λE* the latent heat flux calculated as a function of *T* and ${R}_{n}$^52^. The energy term (${H}^{{\prime}}-{H}_{p}^{{\prime}}$) represents, therefore, the short-term potential of soil moisture to affect *T* through changes in the partitioning of the available radiative energy. When soil moisture is sufficient to meet the atmospheric demand for water, evaporation equals the potential evaporation, and the energy term is zero. Under dry conditions, as atmospheric water demand increases and soil moisture gradually decreases, the energy term increases. Ultimately the soil moisture–temperature coupling (π) will be high when positive values of ${T}^{{\prime}}$ concur with high levels of (${H}^{{\prime}}-{H}_{p}^{{\prime}}$). This method was developed and validated by Ref.^9^. Since then, it has been widely used in many published studies focusing on different regions of the globe and where the soil moisture–temperature coupling conditions were assessed through different perspectives (e.g.^6,53,54^).

### Contribution of temperature advection and radiative processes to the near-surface temperature variation

The near-temperature variation for each grid-cell can be determined by the contribution of the temperature advection (horizontally and vertically) and local radiative processes using a fixed space (point-by-point) Eulerian approach.

3 $${\left(\frac{\Delta T}{\Delta t}\right)}_{h}\left(\lambda ,\phi ,t\right)= -\overrightarrow{v}\cdot {\nabla }_{p}T,$$

4 $${\left(\frac{\Delta T}{\Delta t}\right)}_{v}\left(\lambda ,\phi ,t\right)= -\omega \frac{T}{\theta }\frac{\partial \theta }{\partial p},$$

where $\lambda ,\phi ,t$ represent latitude, longitude and time, respectively, $v$ indicates horizontal wind speed, $T$ temperature, $\omega$ vertical velocity and $\theta$ potential temperature. The temperature advection by the horizontal wind can be calculated by (3), while (4) represents the temperature advection by vertical motion. Temperature changes due to sensible heat advected from remote regions^12^ are, according to this Eulerian approach, comprised in the horizontal and vertical temperature advection terms. Both contributions were computed at an hourly scale, in constant pressure coordinates, and according to particular pressure levels available in the ERA5 reanalysis datasets that correspond to the local near-surface atmospheric layer (from 950 to 900 hPa). The temperature change rate due to diabatic processes, including local sensible heat fluxes induced by local soil desiccation, was estimated as a residual from the previous two terms based on the temperature tendency Eq. (5):

5 $${\left(\frac{\Delta T}{\Delta t}\right)}_{d}\left(\lambda ,\phi ,t\right)= \frac{\Delta T}{\Delta t}-{\left(\frac{\Delta T}{\Delta t}\right)}_{h}-{\left(\frac{\Delta T}{\Delta t}\right)}_{v}.$$

The determination of the diabatic process as a residual term involves some careful considerations. Different factors, such as sub-grid turbulent mixing, analysis increments or even other numerical errors, may contribute to this residual term^55^. This analysis was performed by computing average values of the ERA5 model grid points located within UASP and UACT (Fig. 1f).

## Supplementary Information

Supplementary Information.

## Data Availability

The datasets generated and analyzed during the current study are available from the corresponding author on reasonable request.
