# Supplementary material for: The influence of soil dry-out on the record-breaking hot 2013/2014 summer in Southeast Brazil
Source: Sci Rep. 2022 Apr 7;12:5836. doi: 10.1038/s41598-022-09515-z (PMC8991262; doi:10.1038/s41598-022-09515-z)
Supplement: Supplementary file 1 — Supplementary Information. [file 41598_2022_9515_MOESM1_ESM.pdf]

Supplementary Material

Introduction

This document provides supporting information about the methods used in the Manuscript and also several figures that are important for a complete and detailed interpretation of the results presented in the Manuscript.

HWMId definition

The HWMId was defined as the maximum magnitude of HWs in the summer season. The magnitude of each HW event is the sum of the magnitude of the consecutive days composing the episode. The daily magnitude is calculated as follow:

$$M_d(T_d) = \begin{cases} \dfrac{T_d - T_{30y25p}}{T_{30y75p} - T_{30y25p}}, & if\ T_d > T_{30y25p} \\ 0 \quad , & if\ T_d \leq T_{30y25p} \end{cases}$$

with T_d being the daily Tmax on the heatwave day *d*, T_{30y25p} and T_{30y75p} the 25th and 75th percentiles, respectively, of the time series composed of 30-year maximum temperatures within the reference period (1981-2010). The HWMId definition here presented, corresponds to a slight adaptation from the one proposed by Ref.[1], where the HWMId was defined as the maximum magnitude of HWs in a year: here we defined it as the maximum magnitude of HWs in a summer season. Theoretically the summer season encompasses the most severe HW events with the highest observed Tmax values. Therefore, we believe that this slight adaptation in the index originally proposed by Ref.[1], does not imply considerable changes in the results.

Segmented linear regression model with breakpoints

The estimation of the generalized linear models with two segmented relationships in the linear predictor presented in the Manuscript's Figure 3, was obtained using the r-

package “segmented” (https://cran.r-project.org/web/packages/segmented/segmented.pdf). The package estimates the slopes and breakpoints along with standard errors. The algorithm corresponds to an iterative procedure describe in Ref.[2]. Hypothesis testing (about the existence of the breakpoint) and confidence intervals are performed via appropriate methods and functions[3,4].

Supplementary Figures

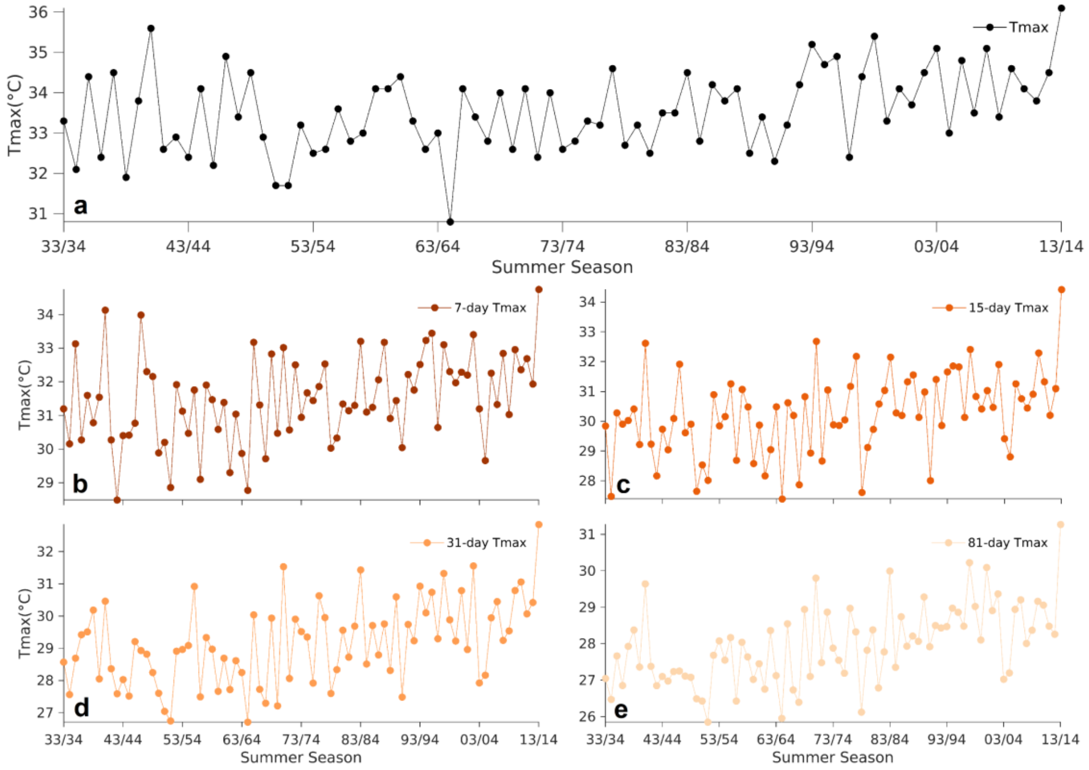

Figure S1. Time series of the mean maximum temperature for the summers between 1933/1934 and 2013/2014 considering raw daily temperature values (**a**), 7-day (**b**), 15-day (**c**), 31-day (**d**) and 81-day (**e**) average periods. These results were obtained using the long-term data from the meteorological station of the University of São Paulo (see Data and Methods).

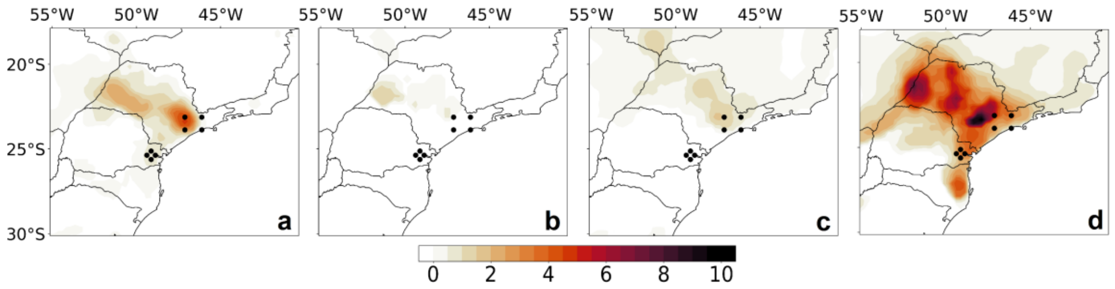

Figure S2. Soil moisture–temperature coupling (π) during the four defined hot periods within the 2013/2014 summer season and chronologically defined in Manuscript's Figure 5e and 5f by the grey boxes.

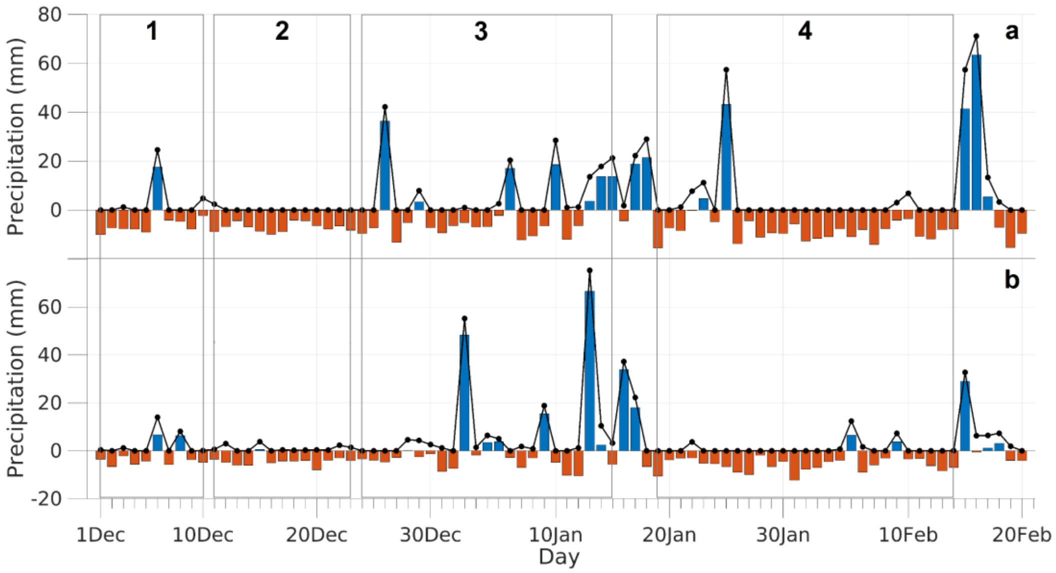

Figure S3. Time series for the 2013/2014 summer season of daily accumulated precipitation (black line) and respective anomaly values (bars) for the UASP (**a**) and UACT (**b**). Results computed using daily precipitation levels recorded from two meteorological stations located in both urban areas (see Data and Methods).

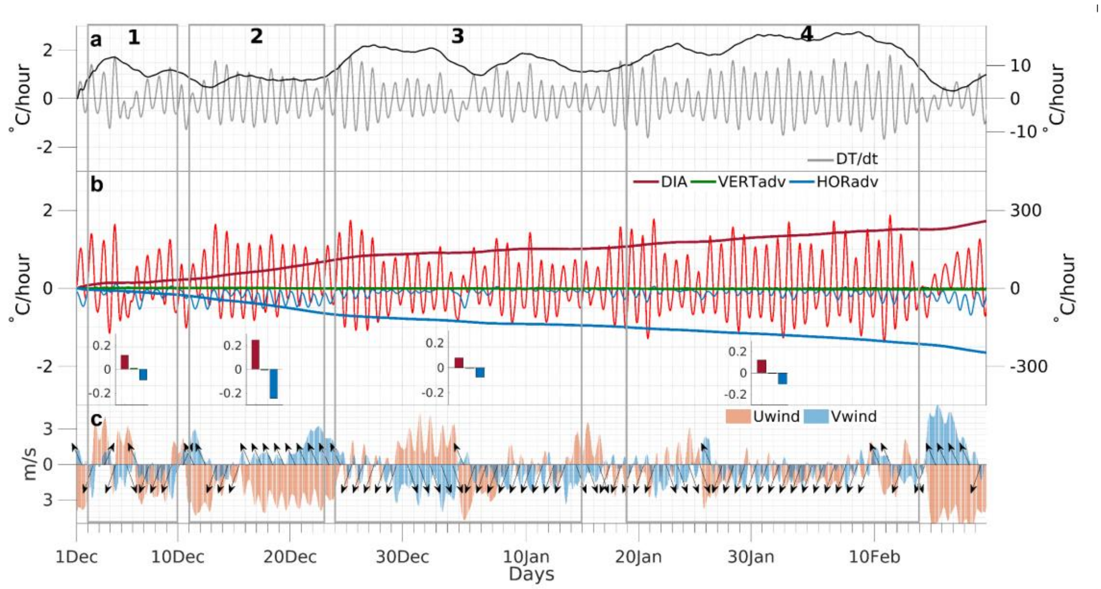

Figure S4. As in Manuscript's Figure 6 but for the UACT.

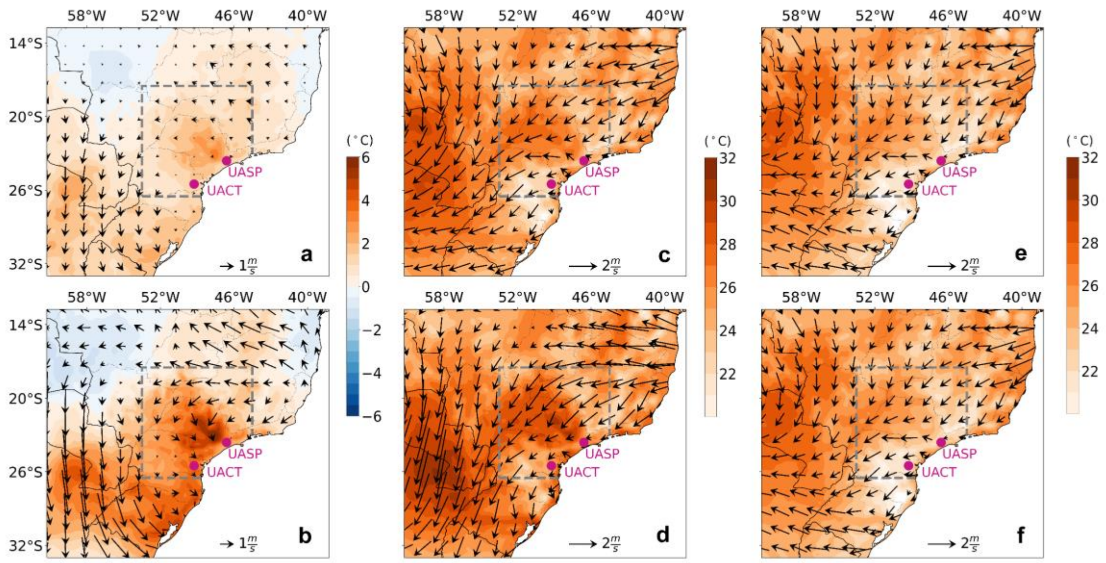

Figure S5. Mean anomaly composite fields over Southeast Brazil of the daily mean surface temperature (ºC, colors) and wind pattern (vectors, m/s) computed for the 2013/2014 summer (**a**) and for the mega-heatwave episode – from January 19th to February 13th 2014 (**b**). Mean observed values over Southeast Brazil of the same meteorological parameters during the 2013/2014 summer (**c**) and during the mega-heatwave episode (**d**). Climatological field (1981–2010 base period) of the same meteorological parameters represented in the previous panels, for the austral summer season (December–February) (**e**) and for the summer period encompassing the mega-heatwave episode (**f**).

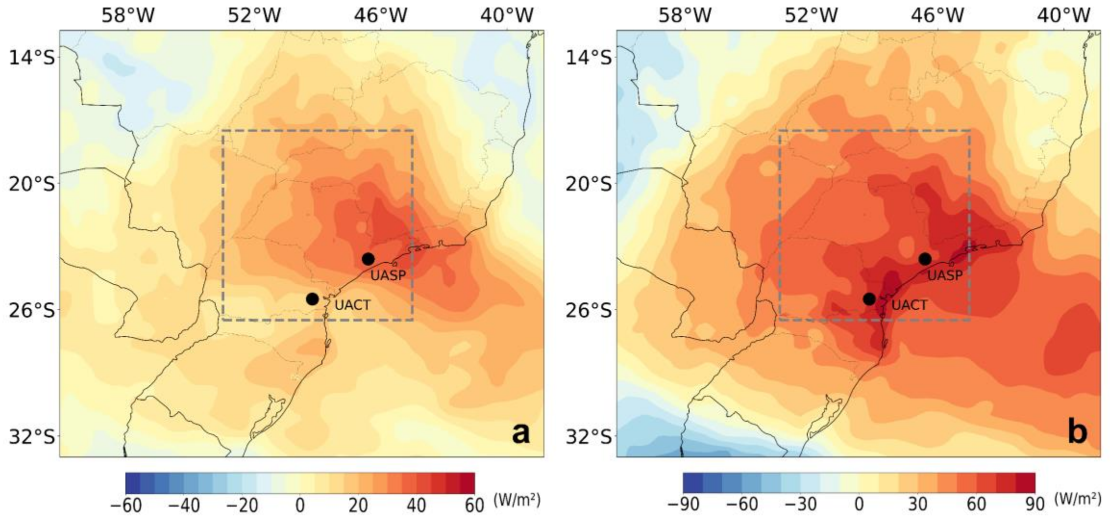

Figure S6. Anomaly composite fields of surface net solar radiation (W/m^2) over Southeast Brazil computed for the 2013/2014 summer (**a**) and for the mega-heatwave episode - from January 19th to February 14th 2014 (**b**).

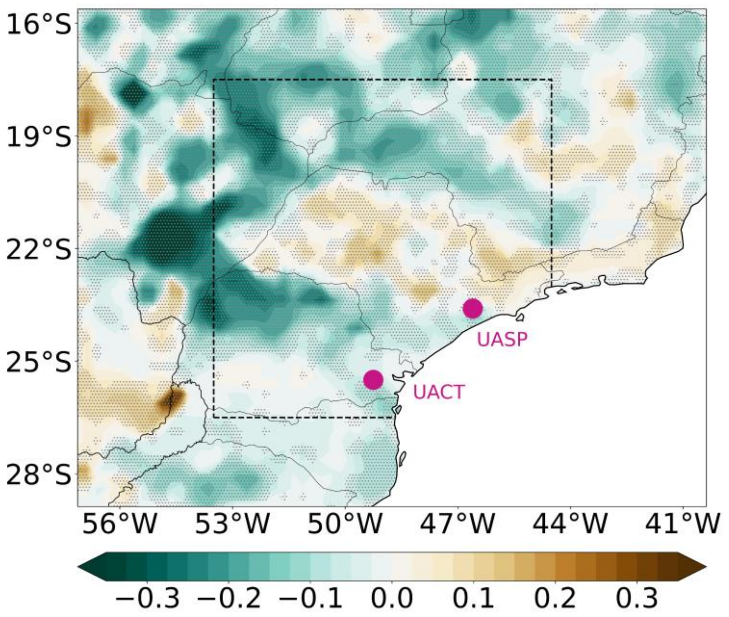

Figure S7. Spatial distribution of correlation coefficient differences between the period encompassing the summers from 2000/2001 to 2019/2020 and the period encompassing the summers from 1980/1981 to 1999/2000. The correlation coefficients were computed between daily soil moisture and evaporative fraction values. The grey dots highlight the grid-points in which the differences were statistically significant at a 5% level

References

1. Russo, S., Sillmann, J. & Fischer, E. M. Top ten European heatwaves since 1950 and their occurrence in the coming decades. *Environ. Res. Lett.* **10**, (2015).

2. Muggeo, V. M. R. Estimating regression models with unknown break-points. *Stat. Med.* **22**, 3055–3071 (2003).

3. Muggeo, V. M. R. Testing with a nuisance parameter present only under the alternative: a score-based approach with application to segmented modelling. *J. Stat. Comput. Simul.* **86**, 3059–3067 (2016).

4. Muggeo, V. M. R. Interval estimation for the breakpoint in segmented regression: a smoothed score-based approach. *Aust. \& New Zeal. J. Stat.* **59**, 311–322 (2017).
